# Virtual Reality-Supported Speech Therapy in Children with Developmental Language Disorder: A Randomized Controlled Trial

**DOI:** 10.3390/medsci14020291

**Published:** 2026-06-05

**Authors:** Carmela De Domenico, Margherita La Fauci, Noemi Mancuso, Mariarita Caputo, Marcella Di Cara, Adriana Piccolo, Alessia Fulgenzi, Daniele Borzelli, Caterina Impallomeni, Emanuela Tripodi, Rocco Salvatore Calabrò, Angelo Quartarone, Francesca Cucinotta

**Affiliations:** 1 IRCCS Centro Neurolesi “Bonino-Pulejo”, Via Provinciale Palermo, Contrada Casazza, 98124 Messina, Italy; carmela.dedomenico@irccsme.it (C.D.D.); noemi.mancuso@irccsme.it (N.M.); mariarita.caputo@irccsme.it (M.C.); marcella.dicara@irccsme.it (M.D.C.); adriana.piccolo@irccsme.it (A.P.); alessia.fulgenzi@irccsme.it (A.F.); caterina.impallomeni@irccsme.it (C.I.); emanuela.tripodi@irccsme.it (E.T.); roccos.calabro@irccsme.it (R.S.C.); angelo.quartarone@irccsme.it (A.Q.); francesca.cucinotta@irccsme.it (F.C.); 2 Laboratory of Physiology, Department of Translational Medicine, Università del Piemonte Orientale, 28100 Novara, Italy; daniele.borzelli@uniupo.it; 3 Laboratory of Neuromotor Physiology, IRCCS Santa Lucia Foundation, 00179 Rome, Italy

**Keywords:** developmental language disorder, virtual reality rehabilitation system, speech therapy intervention, neurodevelopmental disorders

## Abstract

Background/Objectives: Digital technologies are increasingly explored as complementary tools in speech and language therapy for children with neurodevelopmental disorders. However, evidence on virtual reality-based interventions for children with developmental language disorder (DLD) remains limited. This study aimed to evaluate the effects of a Virtual Reality Rehabilitation System (VRRS)-based language intervention combined with standard speech therapy in preschool children with DLD. Secondary objectives included assessing the feasibility, usability, and safety of the VRRS-integrated intervention. Methods: A randomized controlled pilot study was conducted in preschool children diagnosed with DLD. Participants were allocated to an experimental group receiving VRRS-based language intervention integrated with conventional therapy or to a control group receiving standard speech therapy alone. Both groups attended two 60 min sessions per week for six months. Clinical language outcomes were assessed at baseline (T0) and post-intervention (T1). Feasibility was evaluated through adherence and retention rates, usability through a therapist-completed questionnaire, and safety through monitoring of adverse events during sessions. Results: All participants in the experimental group completed the intervention (100% retention). No adverse events were observed. Therapists reported good usability of the VRRS system, highlighting ease of exercise customization, intuitive monitoring of progress, and good integration into routine therapy. Conclusions: VRRS-based activities integrated into conventional speech therapy appear feasible, safe, and well accepted in preschool children with DLD. Further controlled studies with larger samples are needed to confirm these findings. Trial Registration: ClinicalTrials.gov (NCT07438639).

## 1. Introduction

Developmental Language Disorder (DLD) is characterized by persistent difficulties in expressive and/or receptive language that are not caused by other medical conditions [1]. This disorder is one of the most prevalent neurodevelopmental disorders, affecting an estimated 7–10% of children, depending on diagnostic criteria and population characteristics [2,3]. The condition is often linked to long-term difficulties in academic performance, social relationships, and emotional well-being, and delayed diagnosis or intervention further increases the risk of internalizing and externalizing problems [4,5]. Although traditional speech and language therapy remains the gold standard, it can present structural and motivational limitations, especially in young children or when therapy access is constrained [6]. These challenges became even more apparent during the COVID-19 pandemic, which disrupted in-person services and underscored the need for flexible, technology-based treatment options [7]. Moreover, conventional therapy often relies on repetitive paper-based exercises that may not sustain children’s attention [8]. In contrast, recent evidence suggests that immersive, play-based environments can enhance engagement and learning outcomes, particularly in younger children [9]. The increasing demand for innovative and motivating approaches in children’s language intervention has led to growing interest in technology-supported therapies [10]. Among these, virtual reality (VR) has emerged as a promising tool to improve two critical challenges in speech and language therapy: clinical effectiveness and therapeutic engagement [11,12]. Virtual reality offers multisensory, interactive, and ecologically valid settings that simulate real-life communication tasks and provide immediate feedback [11,13]. Furthermore, emerging findings suggest that immersive virtual environments may have a powerful effect on younger children, who tend to exhibit greater neural and behavioral responsiveness to multisensory and feedback-rich contexts [9]. Indeed, immersive and multisensory environments have been shown to enhance attention, working memory, and sensorimotor integration—processes essential for lexical and syntactic learning [14,15,16]. Moreover, VR platforms provide immediate feedback and opportunities for error-based learning, which may facilitate neural plasticity within language-related networks, particularly in young children, whose cognitive flexibility is still developing [17]. These neurocognitive mechanisms support the rationale for integrating VR technologies into child speech and language rehabilitation [18,19].

Among the various digital rehabilitation tools, the Virtual Reality Rehabilitation System (VRRS, Khymeia, Padova, Italy) has shown promising applications in pediatric populations with neurodevelopmental disorders, including cerebral palsy and other developmental conditions [13,20,21]. Several studies using the VRRS have reported improvements in task engagement, attention, and cognitive–linguistic skills, attributed to system features such as real-time feedback and adjustable difficulty levels [22,23]. Similar enhancements in engagement and executive functioning have also been observed in other immersive virtual rehabilitation environments [24,25].

Building on these findings, the present study aimed to replicate and extend our previous preliminary results by increasing sample size and statistical power. We hypothesized that VR-based intervention, combined with standard therapy, may improve language development in children aged 3 to 7 years with DLD. As secondary outcomes, feasibility, usability, and safety were evaluated. We also explored whether individual differences in developmental level could influence responsiveness to VR-based language therapy. Clarifying this relationship could help tailor digital interventions to children most likely to benefit.

## 2. Materials and Methods

### 2.1. Study Design

This randomized, single-blind, controlled trial aimed to evaluate the feasibility and efficacy of a virtual reality-based language intervention for children with DLD, completing a previously published pilot study [1]. The current study increases the original sample while maintaining the same design and treatment protocol used in the pilot phase. The study was conducted at the IRCCS Centro Neurolesi “Bonino Pulejo” in Messina, Italy, between February 2021 and July 2025. Ethics approval was obtained from the IRCCS “Bonino-Pulejo” Local Ethics Committee (Protocol No. 15/2019). The study was registered on ClinicalTrials.gov (Identifier: NCT07438639). The study was performed in accordance with the Declaration of Helsinki [26]. Written informed consent was required from parents or legal guardians for all participants. In addition, children aged 6 years and older provided their consent. All procedures adhered to the CONSORT 2025 guidelines for randomized trials [27].

### 2.2. Participants

Eligible participants were children aged between 3 and 7 years diagnosed with DLD according to DSM-5 [28] criteria, with an adequate level of development, defined as a Developmental Quotient ≥ 85 on the Griffiths Scales of Child Development, Third Edition (Griffiths III; GMS) [29]. Other inclusion criteria were the absence of neurological, sensory, or other psychiatric conditions. Additionally, written informed consent was required from parents or legal guardians, and from children aged ≥ 6 years. Children were excluded if they did not meet the diagnostic criteria for DLD, fell outside the specified age range, had a Global Developmental Delay diagnosis or a co-occurring neurological, sensory, or psychiatric disorders; moreover, the absence of informed consent was a reason for exclusion. Participants who met these criteria were randomly assigned to one of two parallel arms, Experimental Group or Control Group (EG or CG), using a computer-generated randomization list. An uninvolved administrator kept the randomization list confidential, and none of the evaluators was aware of the type of intervention the children were undergoing.

### 2.3. Neuropsychological and Language Assessment

All participants underwent a comprehensive clinical and neuropsychological evaluation conducted by a multidisciplinary team composed of pediatric neuropsychiatrists, psychologists, and speech-language therapists. The diagnosis of DLD was confirmed through multidisciplinary clinical evaluation, integrating clinical observation, structured play sessions, parental interviews, and the Test of Language Development (TVL) [30], a standardized Italian assessment tool for preschool-aged children [31,32,33].

All participants underwent an otolaryngological evaluation, including hearing threshold assessment, to exclude hearing impairments. In addition, other neurodevelopmental conditions that could present similar symptoms were excluded during the diagnostic process. Children’s global development was assessed using the Griffiths III Scales, which evaluate multiple domains of development (such as motor, language, and personal–social skills). The Developmental Quotient (DQ) was used as an overall index of developmental progress and considered as an inclusion criterion. According to the Griffiths Scales, a score ≥ 85 was considered within the normal range. The DQ was also examined as a potential moderator in subsequent analyses.

### 2.4. Outcome Measures

The primary outcome measure was the TVL. The TVL, through 14 items, assesses four main domains of language functioning: receptive language (picture-based comprehension tasks), sentence repetition (15 syntactically graded sentences), naming (body parts and common objects), and spontaneous language production (narration and description of scenes) [34,35]. In Table 1, all TVL subscales were reported and described.

Language skills were evaluated at baseline (T0) and post-treatment (T1), and the primary outcome was defined as the change in TVL total and subscale scores from baseline (T0) to post-intervention (T1), representing the overall improvement in language functioning. Outcome measures included feasibility, usability, and safety. Feasibility was assessed via adherence to the VRRS intervention protocol, quantified by session attendance, completion of planned activities, and progression in task difficulty levels. Usability of the VRRS system was evaluated using a structured therapist-completed questionnaire consisting of 12 items rated on a 5-point Likert scale (1 = not true at all; 5 = very much true). The questionnaire was developed for the purposes of this study and was not a standardized instrument. It addressed ease of use, integration into routine therapy, exercise customization, and perceived child engagement during VR-based language activities. The full questionnaire is provided in Table 2. Safety was monitored through systematic recording by of clinical observation of signs of discomfort, visual fatigue, or behavioral reactions during or after VRRS sessions, using behavioral data collection procedures indicating the presence or absence of target behaviors.

To verify the influence of individual developmental differences, the DQ of GMS was also used as a potential moderator in subsequent analyses. All the assessments were administered by 4 evaluators with several years’ experience in the field, who were qualified neuropsychomotor therapists (n.2) or speech therapists (n.2). The assessments were performed at baseline (T0) and after intervention (T1). All the raters were blind to the type of intervention the children underwent. Despite the impossibility of a formal double-blinding design, these measures minimized the risk of inferring group allocation by assessors, as the possibility of comparing treatment responses and adverse effects between groups was remote.

### 2.5. Intervention

All participants received two 60 min speech therapy sessions per week for six months. Both treatment arms addressed the same core language domains (comprehension, repetition, naming, spontaneous production) but differed in the delivery format. The CG received traditional in-person speech-language therapy, involving structured tasks with printed materials, games, and verbal interaction. The EG received the same therapeutic content delivered via the VRRS developed by Khymeia (Padova, Italy). As in the pilot study [1], the VRRS was used in a non-immersive mode, allowing children to interact with exercises through a touchscreen. Activities were personalized in terms of difficulty and duration based on each child’s baseline profile and therapeutic goals. Examples of tasks included picture naming, sentence repetition, and comprehension exercises involving visual stimuli, with immediate visual and auditory feedback provided during task execution. All treatment sessions were delivered by qualified speech-language therapists who developed individualized therapy plans and ensured consistency across participants by adhering to a standardized intervention framework. For further details, readers may refer to the equivalent table that summarizes the language exercises in the previously published study [1].

### 2.6. Statistical Analysis

Data were analyzed using built-in functions in Matlab R2023b (MathWorks, Natick, MA, USA). To assess the effect of the treatment, whether experimental or traditional, a non-parametric paired Wilcoxon signed-rank test (Matlab function ‘signrank’) was performed to compare scores at T1 with respect to T0. Data were initially analyzed on the full sample and subsequently stratified by sex (females and males) to explore potential gender-related differences. In contrast, to evaluate the improvement in scores for patients who underwent the experimental treatment compared to those who received the traditional treatment, a non-parametric unpaired Wilcoxon rank-sum test (Matlab function ‘ranksum’) was applied to compare scores at T1 between patients treated with the experimental or traditional approaches. Additionally, a Wilcoxon rank-sum test was conducted to assess whether there was a significant difference at T0 between patients enrolled in the experimental treatment group and those in the traditional treatment group. Furthermore, the effects of the patient’s age and the Griffiths Developmental Quotient were investigated as potential moderators of the rehabilitation approach for each score. Two separate ANOVAs were conducted: one with rehabilitation type and age as factors, including their interaction, and another with rehabilitation type and the Griffiths Developmental Quotient as factors, also including their interaction. The same analytical approach was applied to the ANOVA models, which were first conducted on the full sample and then repeated separately for females and males to assess potential sex-related differences. However, due to the limited number of female participants, the Griffith Developmental Quotient was not analyzed in the female subgroup. Statistical significance was assessed using the *p*-value, with a *p*-value < 0.05 indicating a statistically significant difference. To fully exploit the randomized controlled design, additional linear mixed-effects models were implemented to directly assess treatment effects. For each outcome variable, models included Rehabilitation Group (experimental vs. traditional), Time (T0 vs. T1), and their interaction (Group × Time) as fixed effects, with Subject included as a random intercept to account for repeated measures within participants. The Group × Time interaction was considered the primary inferential test of differential treatment effects between rehabilitation approaches. In addition, effect sizes for non-parametric analyses were computed as $r=Z/\sqrt{N}$ for Wilcoxon signed-rank and rank-sum tests. Bootstrap resampling (1000 iterations) was used to estimate 95% confidence intervals for median differences between conditions. For ANOVA models, effect sizes were quantified using partial eta squared (η^2^).

## 3. Results

**Figure 1:**
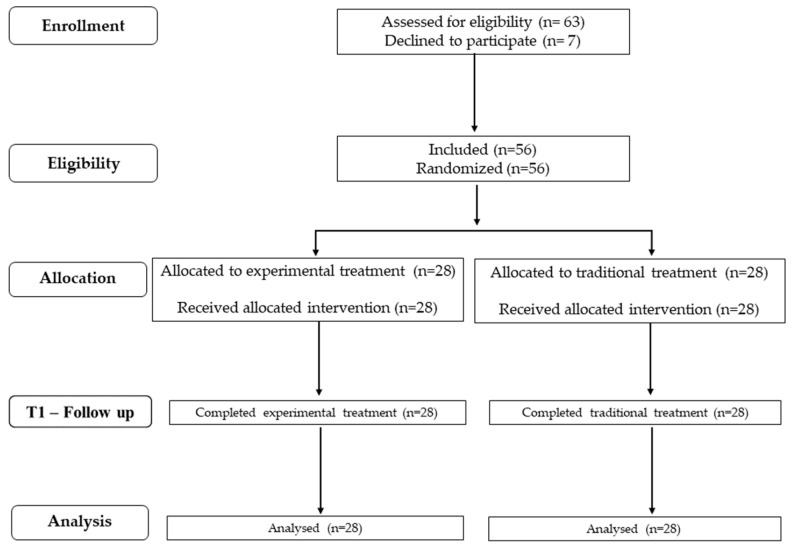
The CONSORT flowchart with detailed information on participants in the study.

A total of 63 potential participants were screened for eligibility from the IRCCS Neurolesi Neuropsychiatry clinic from 01 February 2022 to 30 December 2024. Of these 63, 7 patients were not included due to parental rejection. The participant flow chart is presented in the CONSORT diagram (Figure 1).

Finally, 56 children with DLD were enrolled in the study (mean age ± SD: 4.5 ± 1.1 years; M:F = 2.3:1). Participants were randomly assigned to either the EG (n = 28) or the CG (n = 28). Both groups were comparable in age and sex distribution. Detailed demographic and language profile characteristics are reported in Table 3.

**Figure 2:**
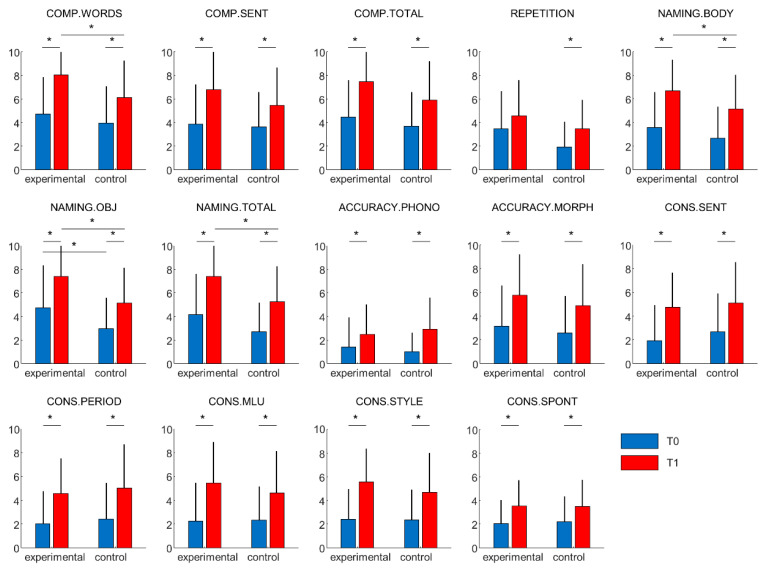
Clinical scores at baseline (T0, blue) and post-treatment (T1, red) for participants in the experimental and control groups. Statistically significant differences are indicated by (*). Bars represent mean values and black lines indicate standard deviations across participants. Abbreviations: COMP.WORDS = computational words; COMP.SENT = sentence comprehension; COMP.TOTAL = total comprehension; NAMING.BODY = naming of body parts; NAMING.OBJ = naming of objects; ACCURACY.PHONO = phonological accuracy; ACCURACY.MORPH = morphosyntactic accuracy; CONS.SENT = sentence construction; CONS.PERIOD = period construction; CONS.MLU = mean length of utterance; CONS.STYLE = construction style; CONS.SPONT = spontaneous production.

A significant improvement in all scores was observed between T0 and T1 for both the experimental and control groups, except for the repetition score in the experimental group. This pattern was consistent both in the full sample (see Table 4 and Figure 2 for a detailed description of the results) and in the male subgroup (Table 5). In contrast, the female subgroup (Table 6) showed a different pattern. In the EG, significant improvements between T0 and T1 were observed in comprehensive words, sentences, and total scores; naming of body parts and total score; and sentence, period, style construction, and mean length of utterance. In the CG, significant improvements were observed only in phonological accuracy, spontaneous production, and style construction. However, despite the lack of a significant difference between experimental and traditional therapy at T0, statistically higher scores were observed at T1 for comprehension of words, body naming, and total naming in the EG whether all participants were considered, and for comprehension of words, naming object and total naming, whether the only male subpopulation was considered. Additionally, a significant improvement in the naming object score was identified at T1 in participants who underwent experimental therapy compared to those who received standard therapy, but this result may be influenced by the statistical difference already present at T0. No effect was observed in the female subpopulation.

As expected, ANOVA showed that children’s age significantly predicted all language scores at T0 (see Table 3), and this developmental trend was maintained at T1 for all measures except repetition, object naming, and total naming. Although no interaction between age and intervention group was observed at T0 for any score, whether the whole population or the male subpopulation was considered, a significant interaction emerged at T1 for comprehension of total words, overall comprehension, repetition, morphosyntactic accuracy, and sentence construction in the whole population and for comprehension of total words in the male subpopulation. The female subpopulation only showed a significant interaction between age and intervention for the computational sentence at T0 but not a T1. Overall, these results indicate that the experimental approach was significantly more effective for younger children. In contrast, ANOVA revealed no effect of the Griffiths Developmental Quotient or its interaction with intervention group on any of the obtained scores neither whether the whole population or the only male subpopulation was considered. Therefore, although both traditional and experimental approaches effectively improved the scores, and therefore patients’ language skills, the greater improvements observed at T1 in some measures suggest that the experimental intervention may be preferable to the traditional one, particularly for younger children. To fully exploit the randomized controlled design, linear mixed-effects models were additionally conducted for each outcome variable, including Rehabilitation Group (experimental vs. control), Time (T0 vs. T1), and their interaction (Group × Time) as fixed effects, with Subject included as a random intercept to account for repeated measures (see Table 7). The Group × Time interaction was considered the primary inferential test of differential treatment effects between rehabilitation approaches. No significant Group × Time interaction was observed for any of the clinical outcomes, indicating that the magnitude of improvement over time did not significantly differ between experimental and control groups when accounting for within-subject variability.

To complement the inferential statistics and provide a quantitative estimate of effect magnitude, Cohen’s d effect sizes with 95% bootstrap confidence intervals were computed for both within-group and between-group comparisons (Table 8). Within-group analyses showed consistent positive effect sizes in both experimental and control groups across most clinical measures, indicating general improvement over time. Between-group comparisons at baseline (T0) confirmed the absence of relevant pre-intervention differences for most outcomes, while post-intervention comparisons (T1) showed variable but generally small-to-moderate differences between groups, consistent with the absence of a significant Group × Time interaction in the mixed-effects models.

### Feasibility, Usability and Safety

**Figure 3:**
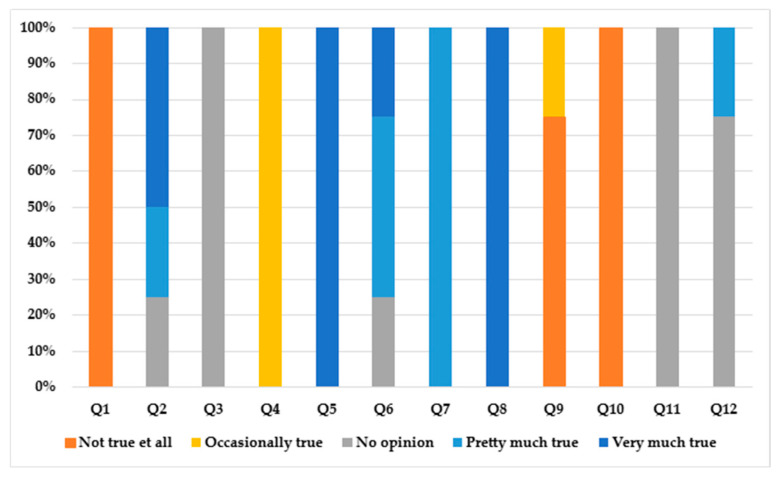
Therapists’ responses to a 12-item VRRS usability questionnaire (5-point Likert scale).

For secondary outcome measures, all participants in the experimental group completed the full set of 48 treatment sessions, corresponding to a 100% retention rate. All planned activities were performed without refusal by participants. No adverse events or signs of discomfort were reported in either group, and no participants withdrew from the study due to adverse reactions. Therapists’ responses to the usability questionnaire are reported in Figure 3. Ratings varied across items and respondents. Minor technical issues were occasionally reported (e.g., software delays, occasional connectivity issues, or temporary system configuration adjustments), which were managed within routine clinical practice without interrupting the therapeutic session. Customization mainly involved task difficulty, number of stimuli, and type of linguistic activity (e.g., naming, repetition). Exercises were generally easy for children to understand, although a brief familiarization phase was occasionally required, particularly in younger participants, without affecting the overall feasibility of the intervention. Monitoring children’s progress within the platform was described as intuitive and manageable during routine therapy sessions. Furthermore, therapists perceived the VRRS exercises as a useful complement to traditional therapy. Overall, therapists expressed a positive attitude toward continued use of the VRRS in clinical speech therapy practice.

## 4. Discussion

This study aimed to extend and verify the findings of a previously published pilot trial by assessing the efficacy of a VR-based language intervention protocol in a larger cohort of pre-school children with DLD. Moreover, secondary goals were to test the feasibility, safety, and usability of the VRRS integrated intervention. Finally, we explored whether responsiveness was influenced by individual differences in developmental level. Limited research exists examining the potential for VR in speech rehabilitation to enable improved communication for children with DLD. Most of the existing literature appears to concern patients with autism or cerebral palsy [13,36,37,38,39,40]. To our knowledge, this is the first randomized, single-blind, controlled study to report on the efficacy, safety, feasibility, and usability of a VR-enhanced speech intervention program for children with DLD. Our results confirm the preliminary findings of the related pilot study. The present study extends previous preliminary findings by increasing the sample size and employing a controlled design, thereby providing more robust evidence on the use of VR-based interventions in children with DLD. Our findings are supported by converging evidence from the pediatric and educational literature, which indicates that immersive and semi-immersive VR contexts improve attention, engagement, and language outcomes through feedback-rich, multisensory interaction. Meta-analytic data on VR-assisted language learning show medium effects on linguistic and affective gains, while a follow-up review of 38 empirical studies (2018–2022) highlights consistent improvements in vocabulary, pronunciation, and narrative skills in VR environments [41,42]. Moreover, Arts et al. [43] reported that interactive VR training enhanced motivation and socio-emotional engagement in adolescents with DLD, reinforcing the potential of immersive contexts to sustain participation and learning during therapy. Although most educational studies involve typically developing students, existing evidence suggested that immersive VR can facilitate word learning and lexicalization, with ERP evidence for improved consolidation during the acquisition of novel words and experimental evidence showing benefits for vocabulary learning and retention in spatially enriched virtual contexts [44,45]. In neurodevelopmental disorders, immersive VR interventions consistently improve social communication and engagement—key factors in treatment adherence—across systematic reviews and mini-reviews, particularly in Autism Spectrum Disorder (ASD) and other Neurodevelopmental Disorders (NDD), strengthening the rationale for VR as an ecologically valid, child-centered context for language rehabilitation [46,47,48]. Complementary pediatric studies indicate that VR-mediated training can strengthen domain-general processes (e.g., executive functions) related to language learning, further supporting transfer to communicative outcomes [49]. In this broader context, our contribution is threefold: we implement a standardized and replicable virtual reality protocol compared to an active control in a clinically homogeneous DLD cohort, addressing design limitations common in previous feasibility studies; we document a developmental characteristic favoring younger children, consistent with theories emphasizing play-based learning and immediate feedback in early childhood [50,51,52,53]. It is important to note that although young children benefit most, all age groups benefit. However, although both traditional and experimental approaches led to improvements in language outcomes, differences between groups were limited to specific measures. As such, the potential added value of the VRRS intervention should be interpreted with caution, and further research with larger samples and longer follow-up is needed to confirm its potential advantages over standard therapy. These findings, combined with previous research, support a shift toward rehabilitation programs in which VR can optimize intensity and adherence while targeting specific language mechanisms (lexical access) within engaging tasks. Future research should test the durability and generalizability of gains, integrate neurophysiological markers to clarify mechanisms, and examine implementation strategies (therapist training, service models) to promote the broader integration of VR-enhanced speech therapy in clinical practice [42].

## 5. Limitations and Future Directions

This study provides promising insights into the application of VR in speech therapy for children with DLD; however, several limitations should be considered when interpreting the findings. Despite the larger sample size compared to the pilot study, an important limitation concerns the inclusion criteria. Although focusing on a homogeneous age range and excluding children with comorbid neurological or psychiatric conditions increased internal validity, it may have restricted the applicability of the findings to children with more complex developmental profiles. Future research should therefore examine whether similar outcomes can be achieved in children with broader neurodevelopmental difficulties, including co-occurring attention, behavioral, or sensory regulation issues. The use of a non-validated therapist-reported questionnaire represents a limitation of the study, as responses may be influenced by subjective bias; however, these findings should be interpreted as exploratory. Due to the nature of the intervention, participants and therapists could not be blinded, which may introduce potential performance and expectation biases. Furthermore, although significant improvements were observed immediately after the intervention, the absence of long-term follow-up prevents conclusions about the maintenance of therapeutic gains. Assessing the persistence of improvements over time would provide valuable information regarding the durability and real-life impact of VR-based interventions. The present study relied primarily on a single standardized language measure, and did not include complementary or ecologically valid outcome measures. Therefore, the extent to which the observed improvements generalize to everyday communication remains to be determined. In addition, the use of the TVL both as part of the diagnostic process and as the primary outcome measure may introduce a potential circularity that should be considered when interpreting the findings. Finally, the reduced number of female participants (17 out of 56) does not allow for a reliable assessment of sex-specific effects of the experimental approach, which could be assessed in future works on a larger population. A further limitation concerns statistical power. The sample size was determined based on feasibility constraints rather than an a priori power analysis. Although the study was likely sufficient to detect moderate within-group changes, it may have been insufficiently powered to detect smaller Group × Time interaction effects, which should therefore be interpreted cautiously.

Finally, we acknowledge that multiple outcome measures were tested, which may increase the risk of Type I error inflation. Given the multidimensional and exploratory nature of the clinical language assessment, no formal correction for multiple comparisons (e.g., Bonferroni or FDR) was applied, as such procedures may increase the risk of Type II error and reduce sensitivity to detect domain-specific effects in heterogeneous clinical profiles. Therefore, results should be interpreted with appropriate caution, with emphasis placed on consistency across outcomes and convergence between analytical approaches (non-parametric tests and mixed-effects models) rather than isolated significant findings.

## 6. Conclusions

The present study adds to the growing evidence supporting the feasibility, safety, and clinical potential of integrating technologies in the clinical practice of pediatric rehabilitation, specifically in children with DLD. It provides a foundation for future research aimed at optimizing, personalizing, and scaling up digital interventions in speech and language therapy. These findings support the integration of virtual reality as a developmentally appropriate, engaging, and potentially beneficial tool for pediatric language rehabilitation. Furthermore, the absence of a significant Group × Time interaction in the linear mixed-effects models suggests that improvements over time were not statistically different between experimental and control groups when accounting for repeated measurements. Therefore, the observed benefits should be interpreted as reflecting a general time-dependent improvement in language performance rather than a treatment-specific differential effect across groups.

## Figures and Tables

**Table 1:** Domain and subitem of TVL.

Index	Domain	Description
**Comprehension words**	Comprehension	Understanding of individual words presented through pictures.
**Comprehension total sentences**	Comprehension	Understanding of orally presented sentences of increasing syntactic complexity.
**Comprehension total**	Comprehension	Global comprehension score combining word and sentence understanding.
**Repetition**	Repetition	Ability to repeat 15 sentences graded for syntactic complexity.
**Naming body**	Naming	Naming of body parts based on visual stimuli.
**Naming objects**	Naming	Naming of common objects presented in pictures.
**Naming total**	Naming	Overall naming performance combining body parts and objects.
**Accuracy phonological**	Accuracy	Phonological accuracy in spontaneous and structured speech.
**Accuracy morphosyntactic**	Accuracy	Morphosyntactic accuracy in sentence production.
**Construction sentence**	Spontaneous production	Ability to construct simple sentences.
**Construction period**	Spontaneous production	Ability to produce complex or compound sentences.
**Construction** **Mean length of utterance**	Spontaneous production	Mean length of utterance in spontaneous speech.
**Construction style**	Spontaneous production	Variety and complexity of syntactic structures used.
**Construction** **Spontaneous production**	Spontaneous production	Overall index of spontaneous language production.

**Table 2:** Therapist-reported VRRS usability questionnaire.

Item	Statement	Domain
1	I experienced difficulties when starting to use the VRRS system.	Ease of use
2	Accessing the VRRS platform and registering each patient was easy.	Ease of use
3	I encountered technical problems while using VRRS.	Technical issues
4	Technical problems interfered with therapy sessions.	Technical issues
5	I felt comfortable using VRRS with all children.	Acceptability
6	Monitoring each child’s progress within VRRS was easy.	Monitoring
7	VRRS exercises were easily customizable according to each child’s needs.	Customization
8	VRRS exercises were easy for children to understand.	Child usability
9	I experienced difficulties using VRRS with children during sessions.	Ease of use
10	The use of VRRS slowed down my clinical workflow.	Workflow
11	VRRS exercises facilitated language rehabilitation compared with standard therapy.	Perceived usefulness
12	I would like to use VRRS more frequently in clinical practice.	Acceptability

**Table 3:** Demographic data of the sample.

	Experimental Group	Control Group	Total
**Enrolled patients (n., %)**	28 (50%)	28 (50%)	56
**Females (n., %)**	10 (35.7%)	7 (25.0%)	17 (30.4%)
**Males (n., %)**	18 (64.3%)	21 (75.0%)	39 (69.6%)
**Age in years (mean ± SD)**	4.4 ± 1.2	4.7 ± 1.1	4.5 ± 1.1
**Greater Expressive impairment**	19 (67.9)	19 (67.9)	38 (67.9)
**Greater Receptive impairment**	0 (0%)	0 (0%)	0 (0%)
**Mixed receptive–expressive impairment**	9 (32.1)	9 (32.1)	18 (32.1)

**Table 4:** Clinical scores at baseline (T0) and post-intervention (T1) for participants in the experimental and control groups.

Clinical Assessment		Experimental Group	*p*-Value (T0 vs. T1)	Control Group	*p*-Value (T0 vs. T1)	*p*-Value (Exp vs. Cont)	*p*-Value (Age)	*p*-Value (Int. Age)	*p*-Value (QS)	*p*-Value (Int. QS)
**COMP.** **WORDS**	**T0**	5.0 (5.0)	<0.001 *, r = 0.74, Δ = 4.0 [1.0–5.0]	3.5 (4.0)	<0.001 *, r = 0.67, Δ = 1.7 [1.0–3.0]	0.355, r = 0.12, Δ = 1.5 [−3.0–1.0]	0.004 *, $\eta^{2}$ = 0.38	0.256, $\eta^{2}$ = 0.12	0.170, $\eta^{2}$ = 0.62	0.477, $\eta^{2}$ = 0.36
	**T1**	9.5 (2.0)		6.0 (5.0)		0.012 *, r = 0.33, Δ = 3.5 [−5.0–−0.5]	0.010 *, $\eta^{2}$ = 0.33	0.043 *, $\eta^{2}$ = 0.23	0.423, $\eta^{2}$ = 0.39	0.738, $\eta^{2}$ = 0.23
**COMP.** **SENT**	**T0**	3.5 (6.0)	<0.001 *, r = 0.69, Δ = 1.7 [1.0–4.7]	3.0 (5.0)	0.002 *, r = 0.57, Δ = 1.7 [1.0–3.4]	0.947, r = 0.01, Δ = 0.5 [−3.0–2.5]	0.004 *, $\eta^{2}$ = 0.39	0.229, $\eta^{2}$ = 0.13	0.078, $\eta^{2}$ = 0.82	0.904, $\eta^{2}$ = 0.14
	**T1**	8.5 (6.5)		5.0 (5.0)		0.152, r = 0.19, Δ = 3.5 [−5.0–0.0]	0.017 *, $\eta^{2}$ = 0.29	0.052, $\eta^{2}$ = 0.22	0.671, $\eta^{2}$ = 0.26	0.851, $\eta^{2}$ = 0.17
**COMP.** **TOTAL**	**T0**	4.0 (5.0)	<0.001 *, r = 067, Δ = 3.5 [1.0–5.0]	3.5 (4.0)	<0.001 *, r = 0.63, Δ = 2.0 [0.5–3.5]	0.369, r = 0.12, Δ = 0.5 [−3.0–1.0]	0.006 *, $\eta^{2}$ = 036	0.230, $\eta^{2}$ = 0.13	0.142, $\eta^{2}$ = 0.67	0.610, $\eta^{2}$ = 0.29
	**T1**	9.0 (5.0)		5.5 (6.0)		0.059, r = 0.25, Δ = 3.5 [−6.0–0.5]	0.023 *, $\eta^{2}$ = 0.27	0.006 *, $\eta^{2}$ = 0.36	0.454, $\eta^{2}$ = 0.37	0.842, $\eta^{2}$ = 0.17
**REPETITION**	**T0**	2.0 (5.0)	0.112, r = 0.30, Δ = 1.0 [0.0–2.0]	1.0 (3.5)	<0.001 *, r = 0.66, Δ = 1.0 [1.0–2.0]	0.073, r = 0.24, Δ = 1.0 [−4.0–1.0]	0.027 *, $\eta^{2}$ = 0.27	0.488, $\eta^{2}$ = 0.07	0.425, $\eta^{2}$ = 0.39	0.164, $\eta^{2}$ = 0.63
	**T1**	4.0 (4.0)		3.0 (3.5)		0.151, r = 0.19, Δ = 1.0 [−4.0–2.0]	0.055, $\eta^{2}$ = 0.22	0.039 *, $\eta^{2}$ = 0.24	0.825, $\eta^{2}$ = 0.18	0.199, $\eta^{2}$ = 0.58
**NAMING.** **BODY**	**T0**	3.0 (5.2)	<0.001 *, r = 0.64, Δ = 3.5 [1.5–5.0]	2.0 (5.5)	<0.001 *, r = 0.62, Δ = 2.0 [1.0–4.0]	0.186, r = 0.18, Δ = 1.0 [−3.0–1.0]	0.002 *, $\eta^{2}$ = 0.45	0.669, $\eta^{2}$ = 0.05	0.211, $\eta^{2}$ = 0.57	0.972, $\eta^{2}$ = 0.09
	**T1**	8.0 (3.0)		5.0 (5.0)		0.049 *, r = 0.26, Δ = 3.0 [−4.5–−0.5]	0.020 *, $\eta^{2}$ = 0.28	0.220, $\eta^{2}$ = 0.13	0.524, $\eta^{2}$ = 0.33	0.408, $\eta^{2}$ = 0.40
**NAMING.** **OBJ**	**T0**	5.5 (5.5)	<0.001 *, r = 0.66, Δ = 1.7 [0.2–3.5]	2.5 (5.7)	<0.001 *, r = 0.72, Δ = 1.5 [1.0–3.0]	0.043 *, r = 0.27, Δ = 3.0 [−5.0–1.5]	0.034 *, $\eta^{2}$ = 0.25	0.251, $\eta^{2}$ = 0.12	0.114, $\eta^{2}$ = 0.72	0.391, $\eta^{2}$ = 0.41
	**T1**	9.0 (4.0)		5.0 (4.0)		0.006 *, r = 0.37, Δ = 4.0 [−5.5–−1.5]	0.120, $\eta^{2}$ = 0.17	0.151, $\eta^{2}$ = 015	0.380, $\eta^{2}$ = 0.42	0.581, $\eta^{2}$ = 0.30
**NAMING.** **TOTAL**	**T0**	3.5 (5.7)	<0.001 *, r = 0.69, Δ = 2.5 [0.5–5.0]	2.0 (5.0)	<0.001 *, r = 0.77, Δ = 2.0 [1.0–3.5]	0.087, r = 0.23, Δ = 1.5 [−4.0–1.5]	0.026 *, $\eta^{2}$ = 0.27	0.228, $\eta^{2}$ = 0.13	0.260, $\eta^{2}$ = 0.52	0.800, $\eta^{2}$ = 0.19
	**T1**	10.0 (4.5)		5.0 (5.0)		0.007 *, r = 0.36, Δ = 5.0 [−6.0–−1.0]	0.186, $\eta^{2}$ = 0.14	0.193, $\eta^{2}$ = 0.14	0.594, $\eta^{2}$ = 0.30	0.441, $\eta^{2}$ = 0.38
**ACCURACY.** **PHONO**	**T0**	0.0 (1.0)	0.011 *, r = 0.48, Δ = 0.7 [0.0–2.0]	0.0 (1.5)	<0.001 *, r = 0.80, Δ = 1.5 [1.0–2.0]	0.971, r = 0.00, Δ = 0.0 [−1.0–0.7]	<0.001 *, $\eta^{2}$ = 0.69	0.764, $\eta^{2}$ = 0.04	0.979, $\eta^{2}$ = 0.07	0.631, $\eta^{2}$ = 0.28
	**T1**	2.0 (3.0)		2.0 (3.0)		0.450, r = 0.10, Δ = 0.0 [−1.0–1.2]	<0.001 *, $\eta^{2}$ = 0.72	0.811, $\eta^{2}$ = 0.03	0.953, $\eta^{2}$ = 0.10	0.439, $\eta^{2}$ = 0.38
**ACCURACY.** **MORPH**	**T0**	2.0 (5.7)	0.002 *, r = 0.60, Δ = 2.0 [0.7–5.0]	1.5 (4.0)	<0.001 *, r = 0.68, Δ = 2.0 [1.0–2.7]	0.577, r = 0.07, Δ = 0.5 [−2.5–2.0]	<0.001 *, $\eta^{2}$ = 0.58	0.234, $\eta^{2}$ = 0.12	0.932, $\eta^{2}$ = 0.12	0.685, $\eta^{2}$ = 0.25
	**T1**	6.0 (7.0)		4.5 (6.5)		0.310, r = 0.13, Δ = 1.5 [−4.0–1.5]	0.004 *, $\eta^{2}$ = 0.38	0.029 *, $\eta^{2}$ = 0.26	0.391, $\eta^{2}$ = 0.41	0.920, $\eta^{2}$ = 0.13
**CONS.** **SENT**	**T0**	0.7 (2.5)	<0.001 *, r = 0.69, Δ = 2.0 [1.0–5.0]	1.5 (4.5)	<0.001 *, r = 0.67, Δ = 2.0 [0.5–3.0]	0.434, r = 0.10, Δ = 0.7 [−1.0–3.0]	<0.001 *, $\eta^{2}$ = 0.71	0.161, $\eta^{2}$ = 0.15	0.623, $\eta^{2}$ = 0.28	0.348, $\eta^{2}$ = 0.44
	**T1**	5.0 (4.5)		5.0 (6.0)		0.711, r = 0.05, Δ = 0.0 [−2.5–2.5]	<0.001 *, $\eta^{2}$ = 0.65	0.048 *, $\eta^{2}$ = 0.23	0.609, $\eta^{2}$ = 0.29	0.169, $\eta^{2}$ = 0.63
**CONS.** **PERIOD**	**T0**	1.0 (3.5)	<0.001 *, r = 0.63, Δ = 2.0 [1.0–4.0]	1.5 (3.0)	0.001 *, r = 0.61, Δ = 2.0 [1.0–4.5]	0.661, r = 0.06, Δ = 0.5 [−1.0–2.0]	<0.001 *, $\eta^{2}$ = 0.49	0.479, $\eta^{2}$ = 0.08	0.236, $\eta^{2}$ = 0.54	0.757, $\eta^{2}$ = 0.22
	**T1**	4.5 (5.0)		5.0 (8.0)		0.723, r = 0.05, Δ = 0.5 [−2.5–3.0]	<0.001 *, $\eta^{2}$ = 0.55	0.126, $\eta^{2}$ = 0.16	0.306, $\eta^{2}$ = 0.48	0.407, $\eta^{2}$ = 0.40
**CONS.** **MLU**	**T0**	1.0 (2.0)	<0.001 *, r = 0.70, Δ = 2.5 [1.0–5.0]	1.0 (4.0)	<0.001 *, r = 0.70, Δ = 2.0 [1.0–3.0]	0.933, r = 0.01, Δ = 0.0 [−1.0–2.0]	<0.001 *, $\eta^{2}$ = 0.64	0.417, $\eta^{2}$ = 0.09	0.819, $\eta^{2}$ = 0.19	0.484, $\eta^{2}$ = 0.35
	**T1**	6.0 (7.0)		4.0 (6.5)		0.322, r = 0.13, Δ = 2.0 [−3.5–1.2]	0.007 *, $\eta^{2}$ = 0.35	0.500, $\eta^{2}$ = 0.07	0.800, $\eta^{2}$ = 0.20	0.717, $\eta^{2}$ = 0.24
**CONS.** **STYLE**	**T0**	2.0 (3.5)	<0.001 *, r = 0.76, Δ = 3.7 [1.2–4.0]	2.0 (3.7)	<0.001 *, r = 0.69, Δ = 1.5 [1.0–3.0]	0.960, r = 0.01, Δ = 0.0 [−1.2–1.2]	<0.001 *, $\eta^{2}$ = 1.20	0.861, $\eta^{2}$ = 0.03	0.483, $\eta^{2}$ = 0.36	0.894, $\eta^{2}$ = 0.14
	**T1**	6.0 (3.5)		5.0 (5.5)		0.235, r = 0.16, Δ = 1.0 [−4.0–1.0]	0.021 *, $\eta^{2}$ = 0.28	0.875, $\eta^{2}$ = 0.03	0.705, $\eta^{2}$ = 0.24	0.475, $\eta^{2}$ = 0.36
**CONS.** **SPONT**	**T0**	2.0 (2.5)	0.005 *, r = 0.53, Δ = 1.7 [1.0–3.0]	2.0 (2.5)	0.004 *, r = 0.55, Δ = 2.0 [1.0–2.0]	0.855, r = 0.02, Δ = 0.0 [−1.0–1.5]	<0.001 *, $\eta^{2}$ = 1.15	0.363, $\eta^{2}$ = 0.10	0.573, $\eta^{2}$ = 0.31	0.769, $\eta^{2}$ = 0.21
	**T1**	3.0 (3.0)		3.5 (3.0)		0.973, r = 0.00, Δ = 0.5 [−1.0–2.0]	<0.001 *, $\eta^{2}$ = 0.68	0.081, $\eta^{2}$ = 0.19	0.479, $\eta^{2}$ = 0.36	0.436, $\eta^{2}$ = 0.38

Values are reported as median (interquartile range). *p*-value (T0 vs. T1) refers to the Wilcoxon signed-rank test comparing scores at T0 and T1 within each group. *p*-value (exp vs. cont) refers to the Wilcoxon rank-sum test comparing the experimental and control groups. *p*-value (age) and *p*-value (QS) indicate the main effects of age and Griffiths Developmental Quotient assessed by ANOVA. *p*-value (int. age) and *p*-value (int. QS) indicate the interaction effects between age or QS and rehabilitation type. * indicates statistical significance (*p* < 0.05). r = effect size for Wilcoxon tests; η^2^ = partial eta squared; Δ = median difference.

**Table 5:** Clinical scores at baseline (T0) and post-intervention (T1) for male participants in the experimental and control groups.

Clinical Assessment		Experimental Group	*p*-Value (T0 vs. T1)	Control Group	*p*-Value (T0 vs. T1)	*p*-Value (Exp vs. Cont)	*p*-Value (Age)	*p*-Value (Int. Age)	*p*-Value (QS)	*p*-Value (Int. QS)
**COMP.** **WORDS**	**T0**	4.5 (4.0)	*p* = 0.001 *, r = −0.77, Δ = 5.00 [1.5–5.5]	4.0 (4.6)	*p* = 0.002 *, r = −0.67, Δ = 2.00 [1.0–4.0]	*p* = 0.681, r = 0.07, Δ = −0.50 [−2.0–3.0]	0.006 *, $\eta^{2}$ = 0.62	0.185, $\eta^{2}$ = 0.23	0.197, $\eta^{2}$ = 0.81	0.564, $\eta^{2}$ = 0.37
	**T1**	4.0 (6.0)		3.0 (4.2)		*p* = 0.029 *, r = 0.35, Δ = −3.50 [0.0–5.0]	0.125, $\eta^{2}$ = 0.27	0.135, $\eta^{2}$ = 0.26	0.742, $\eta^{2}$ = 0.24	0.744, $\eta^{2}$ = 0.24
**COMP.** **SENT**	**T0**	4.0 (5.0)	*p* = 0.008 *, r = −0.63, Δ = 3.50 [0.8–5.0]	3.0 (4.4)	*p* = 0.003 *, r = −0.66, Δ = 2.00 [1.0–4.0]	*p* = 0.921, r = 0.02, Δ = −1.00 [−2.8–3.0]	0.003 *, $\eta^{2}$ = 0.72	0.406, $\eta^{2}$ = 0.14	0.153, $\eta^{2}$ = 0.92	0.848, $\eta^{2}$ = 0.17
	**T1**	2.0 (6.5)		1.0 (4.0)		*p* = 0.127, r = 0.24, Δ = −3.00 [−1.0–5.0]	0.070, $\eta^{2}$ = 0.33	0.339, $\eta^{2}$ = 0.16	0.933, $\eta^{2}$ = 0.11	0.678, $\eta^{2}$ = 0.29
**COMP.** **TOTAL**	**T0**	3.0 (6.5)	*p* = 0.008 *, r = −0.63, Δ = 4.00 [1.8–5.5]	2.0 (3.9)	*p* = 0.002 *, r = −0.67, Δ = 2.00 [1.0–4.0]	*p* = 0.436, r = 0.12, Δ = −1.00 [−2.0–4.0]	0.005 *, $\eta^{2}$ = 0.63	0.364, $\eta^{2}$ = 0.15	0.179, $\eta^{2}$ = 0.85	0.574, $\eta^{2}$ = 0.36
	**T1**	4.0 (6.0)		3.0 (5.5)		*p* = 0.128, r = 0.24, Δ = −3.00 [−2.0–5.8]	0.108, $\eta^{2}$ = 0.29	0.049 *, $\eta^{2}$ = 0.38	0.770, $\eta^{2}$ = 0.23	0.687, $\eta^{2}$ = 0.28
**REPETITION**	**T0**	3.0 (6.5)	*p* = 0.082, r = −0.41, Δ = 1.50 [0.0–3.5]	2.0 (4.6)	*p* = 0.001 *, r = −0.69, Δ = 1.00 [1.0–2.0]	*p* = 0.225, r = 0.19, Δ = −1.00 [−2.0–4.5]	0.031 *, $\eta^{2}$ = 0.43	0.532, $\eta^{2}$ = 0.11	0.277, $\eta^{2}$ = 0.66	0.439, $\eta^{2}$ = 0.47
	**T1**	0 (4.0)		0.5 (2.0)		*p* = 0.094, r = 0.27, Δ = −3.00 [−1.5–4.0]	0.125, $\eta^{2}$ = 0.27	0.094, $\eta^{2}$ = 0.30	0.909, $\eta^{2}$ = 0.13	0.222, $\eta^{2}$ = 0.76
**NAMING.** **BODY**	**T0**	1.5 (4.0)	*p* = 0.010 *, r = −0.61, Δ = 4.25 [2.0–6.0]	1.0 (4.0)	*p* = 0.002 *, r = −0.69, Δ = 2.00 [1.0–5.0]	*p* = 0.287, r = 0.17, Δ = −1.00 [−1.5–4.5]	0.026 *, $\eta^{2}$ = 0.44	0.820, $\eta^{2}$ = 0.05	0.399, $\eta^{2}$ = 0.51	0.989, $\eta^{2}$ = 0.05
	**T1**	0.2 (2.0)		1.0 (6.0)		*p* = 0.103, r = 0.26, Δ = −3.00 [−1.0–5.0]	0.080, $\eta^{2}$ = 0.32	0.272, $\eta^{2}$ = 0.19	0.500, $\eta^{2}$ = 0.42	0.173, $\eta^{2}$ = 086
**NAMING.** **OBJ**	**T0**	1.0 (4.0)	*p* = 0.004 *, r = −0.67, Δ = 3.00 [0.2–6.0]	2.0 (3.2)	*p* = 0.001 *, r = −0.73, Δ = 1.50 [1.0–3.0]	*p* = 0.221, r = 0.20, Δ = −1.00 [−3.5–5.0]	0.246, $\eta^{2}$ = 0.20	0.440, $\eta^{2}$ = 0.13	0.238, $\eta^{2}$ = 0.73	0.755, $\eta^{2}$ = 0.24
	**T1**	1.0 (3.5)		1.0 (5.0)		*p* = 0.021 *, r = 0.37, Δ = −3.00 [1.0–6.0]	0.445, $\eta^{2}$ = 0.13	0.083, $\eta^{2}$ = 0.32	0.467, $\eta^{2}$ = 0.45	0.487, $\eta^{2}$ = 0.43
**NAMING.** **TOTAL**	**T0**	2.0 (5.0)	*p* = 0.004 *, r = −0.68, Δ = 3.50 [0.5–7.0]	2.0 (3.9)	*p* < 0.001 *, r = −0.81, Δ = 2.00 [1.0–4.0]	*p* = 0.281, r = 0.17, Δ = −1.00 [−3.0–5.0]	0.128, $\eta^{2}$ = 0.27	0.673, $\eta^{2}$ = 0.08	0.321, $\eta^{2}$ = 0.60	0.866, $\eta^{2}$ = 0.16
	**T1**	1.5 (3.5)		2.0 (3.5)		*p* = 0.019 *, r = 0.38, Δ = −4.00 [0.5–7.0]	0.258, $\eta^{2}$ = 0.19	0.064, $\eta^{2}$ = 0.34	0.595, $\eta^{2}$ = 0.35	0.309, $\eta^{2}$ = 0.62
**ACCURACY.** **PHONO**	**T0**	9.5 (2.0)	*p* = 0.019 *, r = −0.55, Δ = 2.00 [0.5–3.0]	6.0 (4.2)	*p* < 0.001 *, r = −0.78, Δ = 1.50 [1.0–2.0]	*p* = 0.964, r = 0.01, Δ = 0.50 [−1.0–2.5]	0.005 *, $\eta^{2}$ = 0.65	0.919, $\eta^{2}$ = 0.03	0.614, $\eta^{2}$ = 0.33	0.320, $\eta^{2}$ = 0.61
	**T1**	9.0 (7.0)		6.0 (4.5)		*p* = 0.465, r = 0.12, Δ = −1.00 [−1.0–3.0]	0.001 *, $\eta^{2}$ = 0.94	0.845, $\eta^{2}$ = 0.05	0.939, $\eta^{2}$ = 0.11	0.279, $\eta^{2}$ = 0.66
**ACCURACY.** **MORPH**	**T0**	9.0 (6.0)	*p* = 0.005 *, r = −0.67, Δ = 3.50 [1.5–5.0]	6.0 (6.0)	*p* = 0.003 *, r = −0.65, Δ = 2.00 [1.0–3.5]	*p* = 0.720, r = 0.06, Δ = −0.50 [−2.5–3.5]	<0.001 *, $\eta^{2}$ = 0.97	0.287, $\eta^{2}$ = 0.18	0.724, $\eta^{2}$ = 0.26	0.492, $\eta^{2}$ = 0.43
	**T1**	6.0 (3.0)		3.0 (3.2)		*p* = 0.125, r = 0.25, Δ = −2.50 [−2.0–6.0]	0.004 *, $\eta^{2}$ = 0.66	0.109, $\eta^{2}$ = 0.29	0.368, $\eta^{2}$ = 0.55	0.690, $\eta^{2}$ = 0.28
**CONS.** **SENT**	**T0**	8.0 (3.0)	*p* = 0.004 *, r = −0.69, Δ = 4.00 [2.0–5.2]	5.0 (5.2)	*p* = 0.002 *, r = −0.66, Δ = 2.00 [1.0–3.0]	*p* = 0.365, r = −0.15, Δ = 0.75 [−4.0–1.0]	<0.001 *, $\eta^{2}$ = 1.15	0.483, $\eta^{2}$ = 0.12	0.394, $\eta^{2}$ = 0.52	0.275, $\eta^{2}$ = 0.67
	**T1**	9.0 (3.0)		6.0 (5.5)		*p* = 0.932, r = −0.01, Δ = −1.00 [−3.5–3.5]	0.001 *, $\eta^{2}$ = 0.82	0.102, $\eta^{2}$ = 0.29	0.777, $\eta^{2}$ = 0.22	0.228, $\eta^{2}$ = 0.75
**CONS.** **PERIOD**	**T0**	10.0 (4.0)	*p* = 0.009 *, r = −0.62, Δ = 3.00 [1.0–5.0]	6.0 (7.0)	*p* = 0.009 *, r = −0.57, Δ = 2.00 [1.0–4.5]	*p* = 0.805, r = −0.04, Δ = 1.00 [−2.8–2.0]	0.008 *, $\eta^{2}$ = 0.58	0.734, $\eta^{2}$ = 0.07	0.197, $\eta^{2}$ = 0.81	0.521, $\eta^{2}$ = 0.40
	**T1**	3.0 (5.0)		2.0 (1.8)		*p* = 1.000, r = 0.00, Δ = −0.50 [−3.5–4.0]	0.020 *, $\eta^{2}$ = 0.47	0.172, $\eta^{2}$ = 0.24	0.238, $\eta^{2}$ = 0.73	0.916, $\eta^{2}$ = 0.12
**CONS.** **MLU**	**T0**	6.5 (6.0)	*p* = 0.007 *, r = −0.64, Δ = 3.50 [0.8–5.2]	4.0 (7.2)	*p* = 0.002 *, r = −0.68, Δ = 2.00 [1.0–3.0]	*p* = 0.852, r = 0.03, Δ = 0.00 [−2.5–2.0]	0.013 *, $\eta^{2}$ = 0.53	0.558, $\eta^{2}$ = 0.10	0.243, $\eta^{2}$ = 0.72	0.182, $\eta^{2}$ = 0.84
	**T1**	6.0 (5.0)		5.0 (7.2)		*p* = 0.245, r = 0.19, Δ = −2.00 [−1.0–4.5]	0.019 *, $\eta^{2}$ = 0.48	0.732, $\eta^{2}$ = 0.07	0.890, $\eta^{2}$ = 0.14	0.810, $\eta^{2}$ = 0.20
**CONS.** **STYLE**	**T0**	5.5 (5.0)	*p* = 0.001 *, r = −0.79, Δ = 4.00 [2.0–5.0]	5.0 (8.0)	*p* = 0.003 *, r = −0.65, Δ = 2.00 [1.0–3.0]	*p* = 0.954, r = −0.01, Δ = 0.00 [−2.5–2.8]	<0.001 *, $\eta^{2}$ = 1.38	0.962, $\eta^{2}$ = 0.02	0.504, $\eta^{2}$ = 0.42	0.752, $\eta^{2}$ = 0.24
	**T1**	6.0 (7.0)		4.0 (5.5)		*p* = 0.099, r = 0.26, Δ = −2.00 [−1.0–5.0]	0.078, $\eta^{2}$ = 0.32	0.528, $\eta^{2}$ = 0.11	0.721, $\eta^{2}$ = 0.26	0.814, $\eta^{2}$ = 0.20
**CONS.** **SPONT**	**T0**	7.0 (3.0)	*p* = 0.029 *, r = −0.51, Δ = 2.25 [0.8–4.0]	5.0 (5.2)	*p* = 0.045 *, r = −0.44, Δ = 1.50 [1.0–2.0]	*p* = 0.909, r = −0.02, Δ = 0.50 [−2.0–2.0]	<0.001 *, $\eta^{2}$ = 1.15	0.656, $\eta^{2}$ = 0.08	0.568, $\eta^{2}$ = 0.37	0.816, $\eta^{2}$ = 0.20
	**T1**	3.5 (4.0)		4.0 (3.0)		*p* = 0.475, r = 0.11, Δ = 0.50 [−2.0–3.0]	0.003 *, $\eta^{2}$ = 0.73	0.361, $\eta^{2}$ = 0.16	0.985, $\eta^{2}$ = 0.05	0.335, $\eta^{2}$ = 0.59

Values are reported as median (interquartile range) (*p*-value (T0 vs. T1) refers to the Wilcoxon signed-rank test comparing scores at T0 and T1 within each group. *p*-value (exp vs. cont) refers to the Wilcoxon rank-sum test comparing the experimental and control groups. *p*-value (age) and *p*-value (QS) indicate the main effects of age and Griffiths Developmental Quotient assessed by ANOVA. *p*-value (int. age) and *p*-value (int. QS) indicate the interaction effects between age or QS and rehabilitation type. * indicates statistical significance (*p* < 0.05). r = effect size for Wilcoxon tests; η^2^ = partial eta squared; Δ = median difference.

**Table 6:** Clinical scores at baseline (T0) and post-intervention (T1) for female participants in the experimental and control groups.

Clinical Assessment		Experimental Group	*p*-Value (T0 vs. T1)	Control Group	*p*-Value (T0 vs. T1)	*p*-Value (Exp vs. Cont)	*p*-Value (Age)	*p*-Value (Int. Age)
**COMP.** **WORDS**	**T0**	5.5 (5.0)	*p* = 0.024 *, r = −0.71, Δ = 1.50 [0.0–5.0]	3.0 (3.7)	*p* = 0.071, r = −0.68, Δ = 1.00 [0.0–2.0]	*p* = 0.404, r = 0.20, Δ = −2.50 [−3.0–6.0]	0.049 *, $\eta^{2}$ = 0.73	0.986, $\eta^{2}$ = 0.00
	**T1**	2.5 (7.0)		5.0 (5.9)		*p* = 0.187, r = 0.32, Δ = −4.00 [−2.0–9.0]	0.050, $\eta^{2}$ = 0.72	0.323, $\eta^{2}$ = 0.23
**COMP.** **SENT**	**T0**	4.5 (5.0)	*p* = 0.006 *, r = −0.87, Δ = 1.00 [1.0–4.5]	5.0 (3.7)	*p* = 0.293, r = −0.40, Δ = 1.00 [−2.0–3.5]	*p* = 0.922, r = 0.02, Δ = 2.50 [−5.0–6.0]	0.118, $\eta^{2}$ = 0.47	0.031 *, $\eta^{2}$ = 0.88
	**T1**	2.5 (5.0)		2.0 (2.7)		*p* = 0.658, r = 0.11, Δ = −3.50 [−4.8–6.8]	0.285, $\eta^{2}$ = 0.26	0.139, $\eta^{2}$ = 0.43
**COMP.** **TOTAL**	**T0**	3.0 (2.0)	*p* = 0.011 *, r = −0.80, Δ = 2.00 [0.5–6.0]	2.0 (6.0)	*p* = 0.223, r = −0.46, Δ = 0.00 [−1.0–3.0]	*p* = 0.767, r = 0.07, Δ = 0.50 [−3.0–5.0]	0.128, $\eta^{2}$ = 0.45	0.285, $\eta^{2}$ = 0.26
	**T1**	5.5 (5.0)		0.5 (4.0)		*p* = 0.245, r = 0.28, Δ = −5.00 [−2.5–7.0]	0.144, $\eta^{2}$ = 0.42	0.163, $\eta^{2}$ = 0.39
**REPETITION**	**T0**	4.5 (4.0)	*p* = 0.932, r = 0.03, Δ = 0.00 [−1.2–1.0]	0.5 (5.7)	*p* = 0.123, r = −0.58, Δ = 1.00 [1.0–2.0]	*p* = 0.254, r = 0.28, Δ = −0.50 [−1.2–5.5]	0.617, $\eta^{2}$ = 0.09	0.591, $\eta^{2}$ = 0.10
	**T1**	0 (1.0)		0 (0.4)		*p* = 0.961, r = 0.01, Δ = −0.50 [−3.0–3.5]	0.950, $\eta^{2}$ = 0.01	0.350, $\eta^{2}$ = 0.21
**NAMING.** **BODY**	**T0**	2.0 (6.5)	*p* = 0.016 *, r = −0.76, Δ = 2.00 [0.5–5.0]	3.0 (3.6)	*p* = 0.293, r = −0.40, Δ = 1.00 [−1.0–5.0]	*p* = 0.765, r = 0.07, Δ = −1.00 [−3.2–4.5]	0.008 *, $\eta^{2}$ = 1.40	0.258, $\eta^{2}$ = 0.28
	**T1**	1.0 (3.0)		2.0 (2.7)		*p* = 0.211, r = 0.30, Δ = −2.50 [−1.5–5.0]	0.300, $\eta^{2}$ = 0.24	0.442, $\eta^{2}$ = 0.16
**NAMING.** **OBJ**	**T0**	1.0 (3.0)	*p* = 0.041 *, r = −0.65, Δ = 1.00 [0.0–3.0]	1.0 (2.8)	*p* = 0.071, r = −0.68, Δ = 1.00 [0.0–3.0]	*p* = 0.084, r = 0.42, Δ = −5.00 [−1.5–6.8]	0.023 *, $\eta^{2}$ = 0.98	0.481, $\eta^{2}$ = 0.14
	**T1**	1.0 (2.0)		2.0 (2.7)		*p* = 0.166, r = 0.34, Δ = −3.00 [−3.0–7.0]	0.306, $\eta^{2}$ = 0.24	0.794, $\eta^{2}$ = 0.04
**NAMING.** **TOTAL**	**T0**	1.5 (2.5)	*p* = 0.018 *, r = −0.75, Δ = 1.50 [0.0–5.5]	2.0 (1.7)	*p* = 0.128, r = −0.58, Δ = 2.00 [−1.0–4.0]	*p* = 0.346, r = 0.23, Δ = −4.00 [−3.0–5.5]	0.073, $\eta^{2}$ = 0.61	0.091, $\eta^{2}$ = 0.55
	**T1**	2.0 (1.5)		2.0 (2.6)		*p* = 0.197, r = 0.31, Δ = −3.50 [−2.8–7.0]	0.247, $\eta^{2}$ = 0.29	0.685, $\eta^{2}$ = 0.07
**ACCURACY.** **PHONO**	**T0**	9.0 (2.0)	*p* = 0.336, r = −0.30, Δ = 0.00 [0.0–1.0]	5.0 (7.2)	*p* = 0.028, r = −0.83, Δ = 2.00 [1.0–5.0]	*p* = 0.688, r = 0.10, Δ = 0.00 [−0.5–1.0]	0.587, $\eta^{2}$ = 0.10	0.563, $\eta^{2}$ = 0.11
	**T1**	7.5 (6.0)		4.0 (6.5)		*p* = 0.079, r = −0.43, Δ = 1.75 [−5.0–0.5]	0.079, $\eta^{2}$ = 0.59	0.736, $\eta^{2}$ = 0.06
**ACCURACY.** **MORPH**	**T0**	9.0 (4.0)	*p* = 0.478, r = −0.22, Δ = 0.25 [−1.0–3.2]	4.0 (5.5)	*p* = 0.046, r = −0.75, Δ = 1.00 [0.0–7.0]	*p* = 0.693, r = 0.10, Δ = 1.00 [−3.0–5.5]	0.259, $\eta^{2}$ = 0.28	0.843, $\eta^{2}$ = 0.03
	**T1**	3.5 (4.0)		3.0 (3.5)		*p* = 0.768, r = −0.07, Δ = 0.00 [−7.0–4.5]	0.291, $\eta^{2}$ = 0.25	0.214, $\eta^{2}$ = 0.32
**CONS.** **SENT**	**T0**	7.5 (3.0)	*p* = 0.044 *, r = −0.64, Δ = 1.00 [0.5–3.5]	5.0 (2.2)	*p* = 0.068, r = −0.69, Δ = 2.00 [0.0–6.0]	*p* = 1.000, r = −0.00, Δ = 1.00 [−3.0–2.5]	0.361, $\eta^{2}$ = 0.20	0.741, $\eta^{2}$ = 0.05
	**T1**	8.0 (8.0)		5.0 (2.0)		*p* = 0.806, r = −0.06, Δ = 1.00 [−5.0–4.5]	0.073, $\eta^{2}$ = 0.61	0.432, $\eta^{2}$ = 0.16
**CONS.** **PERIOD**	**T0**	8.5 (8.0)	*p* = 0.045 *, r = −0.63, Δ = 2.00 [0.8–5.0]	5.0 (2.5)	*p* = 0.058, r = −0.72, Δ = 4.50 [0.0–5.0]	*p* = 0.763, r = −0.07, Δ = 0.00 [−3.0–2.0]	0.673, $\eta^{2}$ = 0.07	0.489, $\eta^{2}$ = 0.14
	**T1**	0.2 (2.0)		2.0 (3.5)		*p* = 0.694, r = −0.10, Δ = 2.50 [−8.0–4.0]	0.030 *, $\eta^{2}$ = 0.89	0.603, $\eta^{2}$ = 0.10
**CONS.** **MLU**	**T0**	5.0 (6.0)	*p* = 0.010 *, r = −0.81, Δ = 2.00 [1.0–6.0]	5.0 (5.7)	*p* = 0.043 *, r = −0.76, Δ = 2.00 [0.0–8.0]	*p* = 0.720, r = −0.09, Δ = 1.00 [−2.5–2.0]	0.011 *, $\eta^{2}$ = 1.28	0.684, $\eta^{2}$ = 0.07
	**T1**	4.0 (4.0)		5.0 (6.0)		*p* = 1.000, r = −0.00, Δ = 0.00 [−7.8–6.5]	0.214, $\eta^{2}$ = 0.32	0.602, $\eta^{2}$ = 0.10
**CONS.** **STYLE**	**T0**	2.5 (5.0)	*p* = 0.020 *, r = −0.73, Δ = 1.25 [0.2–5.0]	5.0 (7.2)	*p* = 0.034 *, r = −0.80, Δ = 1.00 [0.5–7.0]	*p* = 0.765, r = 0.07, Δ = 0.50 [−1.5–2.0]	0.299, $\eta^{2}$ = 0.24	0.518, $\eta^{2}$ = 0.13
	**T1**	5.0 (7.0)		5.0 (9.0)		*p* = 1.000, r = −0.00, Δ = 1.50 [−6.2–4.0]	0.052, $\eta^{2}$ = 0.71	0.852, $\eta^{2}$ = 0.03
**CONS.** **SPONT**	**T0**	3.5 (5.0)	*p* = 0.067, r = −0.58, Δ = 1.25 [0.0–2.0]	5.0 (7.1)	*p* = 0.034 *, r = −0.80, Δ = 1.00 [0.5–7.0]	*p* = 1.000, r = −0.00, Δ = 0.00 [−1.8–2.0]	0.113, $\eta^{2}$ = 0.48	0.451, $\eta^{2}$ = 0.15
	**T1**	2.5 (2.0)		3.0 (2.0)		*p* = 0.273, r = −0.27, Δ = 0.50 [−3.0–1.0]	0.332, $\eta^{2}$ = 0.22	0.220, $\eta^{2}$ = 0.32

Values are reported as median (interquartile range). *p*-value (T0 vs. T1) refers to the Wilcoxon signed-rank test comparing scores at T0 and T1 within each group. *p*-value (exp vs. cont) refers to the Wilcoxon rank-sum test comparing the experimental and control groups. *p*-value (age) and *p*-value (QS) indicate the main effects of age and Griffiths Developmental Quotient assessed by ANOVA. *p*-value (int. age) and *p*-value (int. QS) indicate the interaction effects between age or QS and rehabilitation type. * indicates statistical significance (*p* < 0.05). r = effect size for Wilcoxon tests; η^2^ = partial eta squared; Δ = median difference.

**Table 7:** Linear mixed-effects model results for clinical outcomes.

Clinical Assessment	T0–T1 Change	Group Effect	Group × Time Interaction
**COMP.WORDS**	<0.001 *	0.344	0.131
**COMP.SENT**	0.002 *	0.771	0.202
**COMP.TOTAL**	<0.001 *	0.354	0.332
**REPETITION**	0.004 *	0.037 *	0.575
**NAMING.BODY**	<0.001 *	0.220	0.493
**NAMING.OBJ**	<0.001 *	0.029 *	0.519
**NAMING.TOTAL**	<0.001 *	0.084	0.379
**ACCURACY.PHONO**	<0.001 *	0.548	0.117
**ACCURACY.MORPH**	0.001 *	0.534	0.740
**CONS.SENT**	<0.001 *	0.381	0.649
**CONS.PERIOD**	<0.001 *	0.616	0.970
**CONS.MLU**	<0.001 *	0.934	0.287
**CONS.STYLE**	<0.001 *	0.962	0.301
**CONS.SPONT**	0.004 *	0.777	0.775

Linear mixed-effects model results for clinical outcomes. Results of linear mixed-effects models including Group (experimental vs. traditional), Time (T0 vs. T1), and their interaction (Group × Time) as fixed effects, with Subject included as a random intercept to account for repeated measures. Values report *p*-values for the main effect of Time (T0–T1 change), Group effect, and Group × Time interaction. Asterisks indicate statistical significance (*p* < 0.05).

**Table 8:** Effect sizes (Cohen’s d) with 95% bootstrap confidence intervals for within- and between-group comparisons.

Clinical Assessment	Within-Group Effect (EG: T1 vs. T0)	Within-Group Effect (CG: T1 vs. T0)	Baseline Group Difference (T0: EG vs. CG)	Post-Treatment Group Difference (T1: EG vs. CG)
**COMP.WORDS**	d = 1.11 [0.76–0.76]	d = 0.80 [0.47–0.47]	d = 0.25 [−0.27–−0.27]	d = 0.63 [0.09–0.09]
**COMP.SENT**	d = 0.82 [0.42–0.42]	d = 0.68 [0.32–0.32]	d = 0.08 [−0.45–−0.45]	d = 0.40 [−0.13–−0.13]
**COMP.TOTAL**	d = 0.91 [0.46–0.46]	d = 0.75 [0.39–0.39]	d = 0.25 [−0.26–−0.26]	d = 0.48 [−0.03–−0.03]
**REPETITION**	d = 0.32 [−0.05–−0.05]	d = 0.86 [0.52–0.52]	d = 0.56 [0.06–0.06]	d = 0.40 [−0.12–−0.12]
**NAMING.BODY**	d = 0.81 [0.39–0.39]	d = 0.80 [0.46–0.46]	d = 0.32 [−0.19–−0.19]	d = 0.55 [0.02–0.02]
**NAMING.OBJ**	d = 0.82 [0.48–0.48]	d = 0.93 [0.65–0.65]	d = 0.57 [0.07–0.07]	d = 0.74 [0.18–0.18]
**NAMING.TOTAL**	d = 0.88 [0.56–0.56]	d = 1.01 [0.74–0.74]	d = 0.47 [−0.03–−0.03]	d = 0.67 [0.14–0.14]
**ACCURACY.PHONO**	d = 0.54 [0.19–0.19]	d = 1.04 [0.75–0.75]	d = 0.18 [−0.40–−0.40]	d = 0.16 [−0.70–−0.70]
**ACCURACY.MORPH**	d = 0.64 [0.25–0.25]	d = 0.70 [0.31–0.31]	d = 0.17 [−0.34–−0.34]	d = 0.25 [−0.27–−0.27]
**CONS.SENT**	d = 0.85 [0.45–0.45]	d = 0.81 [0.53–0.53]	d = 0.23 [−0.80–−0.80]	d = 0.11 [−0.66–−0.66]
**CONS.PERIOD**	d = 0.70 [0.31–0.31]	d = 0.73 [0.36–0.36]	d = 0.14 [−0.70–−0.70]	d = 0.13 [−0.66–−0.66]
**CONS.MLU**	d = 0.87 [0.50–0.50]	d = 0.85 [0.55–0.55]	d = 0.02 [−0.59–−0.59]	d = 0.24 [−0.28–−0.28]
**CONS.STYLE**	d = 1.06 [0.70–0.70]	d = 0.74 [0.45–0.45]	d = 0.01 [−0.53–−0.53]	d = 0.28 [−0.25–−0.25]
**CONS.SPONT**	d = 0.55 [0.15–0.15]	d = 0.65 [0.23–0.23]	d = 0.08 [−0.60–−0.60]	d = 0.01 [−0.53–−0.53]

Effect sizes (Cohen’s d) with 95% bootstrap confidence intervals for within- and between-group comparisons. Within-group effects represent pre-to-post changes (T1 vs. T0) in the experimental and control groups, respectively. Between-group effects represent differences between experimental and control groups at baseline (T0) and post-intervention (T1). Positive values indicate higher scores or greater improvement in the experimental group or at follow-up relative to baseline depending on the comparison.

## Data Availability

The data presented in this study are available on request from the corresponding author due to privacy restrictions.

## Abbreviations

The following abbreviations are used in this manuscript:

DLD	Developmental Language Disorder
VR	Virtual Reality
VRRS	Virtual Reality Rehabilitation System
DSM	Diagnostic and Statistical Manual of Mental Disorders
EG	Experimental Group
CG	Control Group
TVL	Development Level Test
DQ	Developmental Quotient
GMS	Griffiths Mental Development Scales
ERP	Event-related potentials
ASD	Autism Spectrum Disorders
NDD	Neurodevelopmental Disorders
